# Supplementary material for: Ethyl Acetate Fraction of Bixa orellana and Its Component Ellagic Acid Exert Antibacterial and Anti-Inflammatory Properties against Mycobacterium abscessus subsp. massiliense
Source: Antibiotics (Basel). 2022 Jun 17;11(6):817. doi: 10.3390/antibiotics11060817 (PMC9220277; doi:10.3390/antibiotics11060817)
Supplement: Supplementary file 1 [file antibiotics-11-00817-s001.zip › antibiotics-1741806-supplementary.pdf]

Supplementary Material

Ethyl Acetate Fraction of *Bixa orellana* and Its Component Ellagic Acid Exert Antibacterial and Anti-inflammatory properties against *Mycobacterium abscessus* subsp. *massiliense*

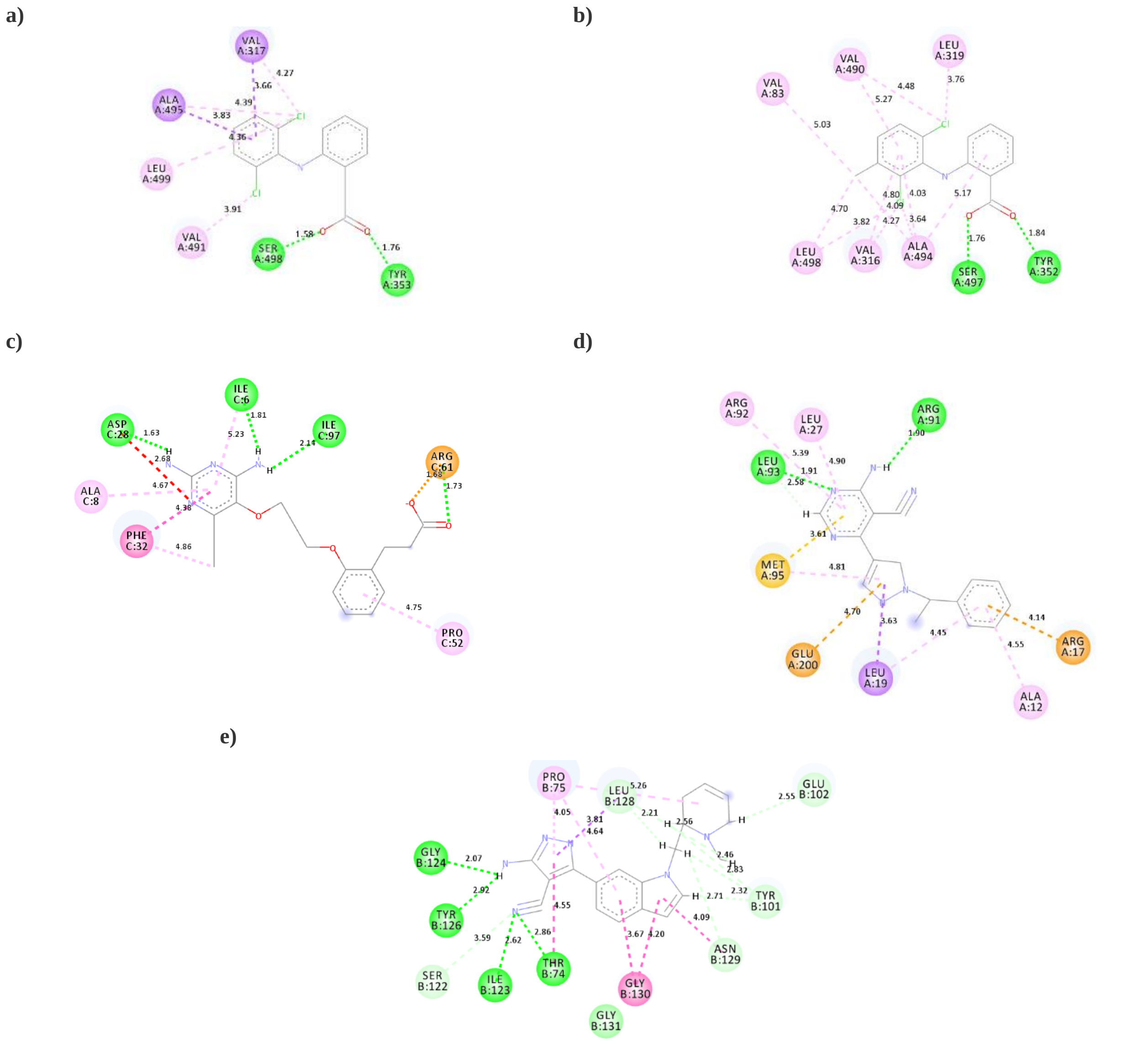

Figure S1: Diagram 2D of Interactions between controls / native ligands and targets: a) diclofenac and COX2 (PDB ID: 1PXX); b) meclofenamic acid and COX2 (PDB ID: 5IKQ); c) MMV ligand and dihydrofolate reductase (PDB ID: 7K6C); d) Q0Q ligand and phosphoribosylaminoimidazole-succinocarboxamide synthase (PDB ID: 6YYB), and e) JD8 ligand and tRNA (guanine-N(1)-)-methyltransferase (PDB ID: 6QR4). Interactions: Conventional Hydrogen Bond: ■, Pi-Sigma: ■, Alkyl/ Pi-Alkyl: ■, Van der Walls: ■, Salt Bridge: ■ (present in c), Pi-Pi stacked: ■ (present in c), Unfavorable Aceptor-Aceptor: ■, Pi-cation: ■ (present in d), Carbon Hydrogen Bond: ■.

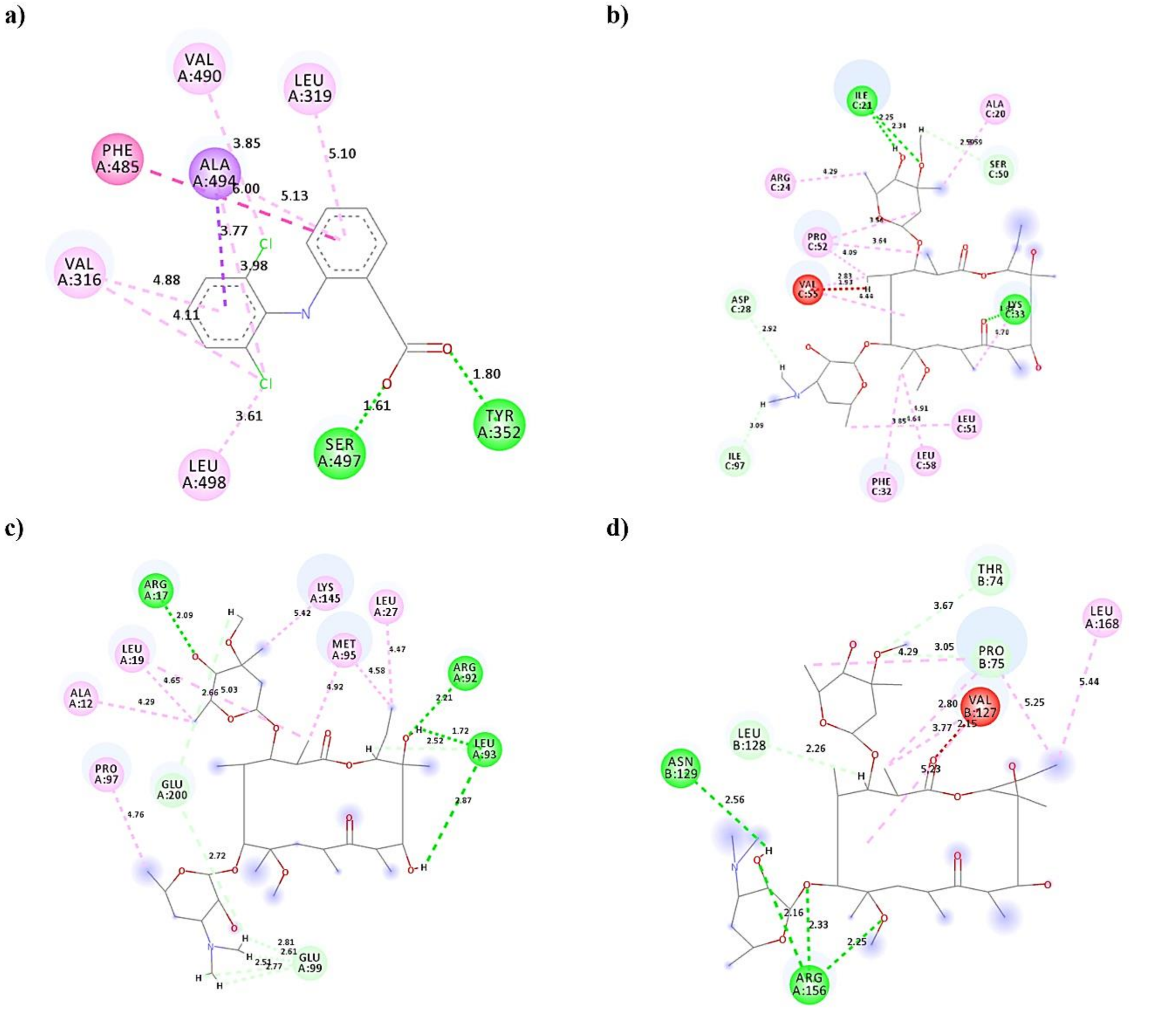

Figure S2: Diagram 2D of Interactions between controls and targets. a) Interactions between diclofenac and COX2 (PDB ID: 5IKQ); b) Interactions between claritromycin and Dihydrofolate reductase (PDB ID 7K6C); c) Interactions between claritromycin and Phosphoribosylaminoimidazole-succinocarboxamide synthase (PDB ID 6YYB); d) Interactions between clarithromycin and tRNA (guanine-N(1)-)-methyltransferase (PDB ID 6QR4).

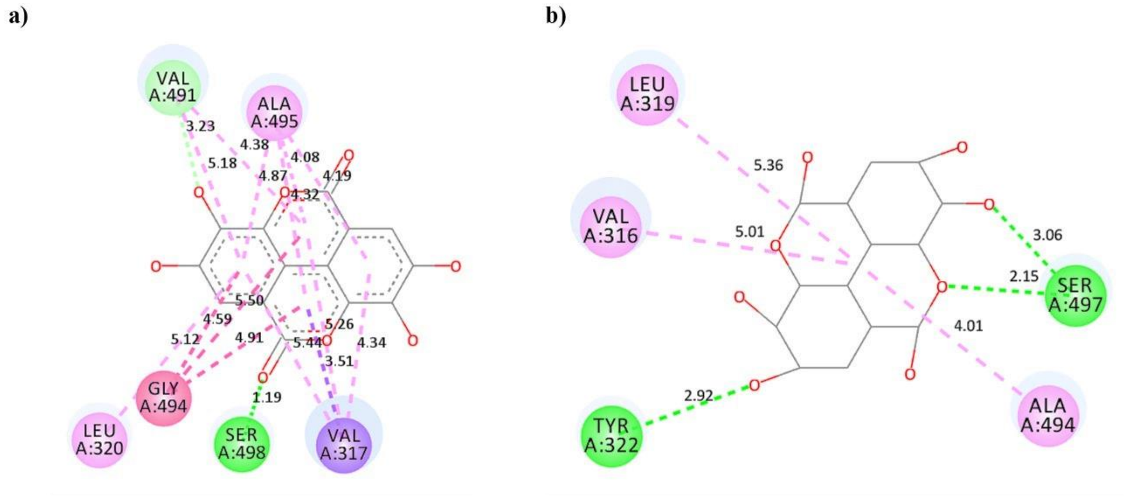

Figure S3: Diagram 2D of Interactions between ellagic acid and COX2 targets: (a) with the murine COX2 (PDB ID: 1PXX) and (b) with the human COX2 (PDB ID: 5IKQ). Interactions: ■ - Conventional Hydrogen Bond, ■ - Pi-Sigma, ■ - Alkyl/ Pi-Alkyl, ■ - Carbon Hydrogen Bond, ■ - Amide Pi-Stacked

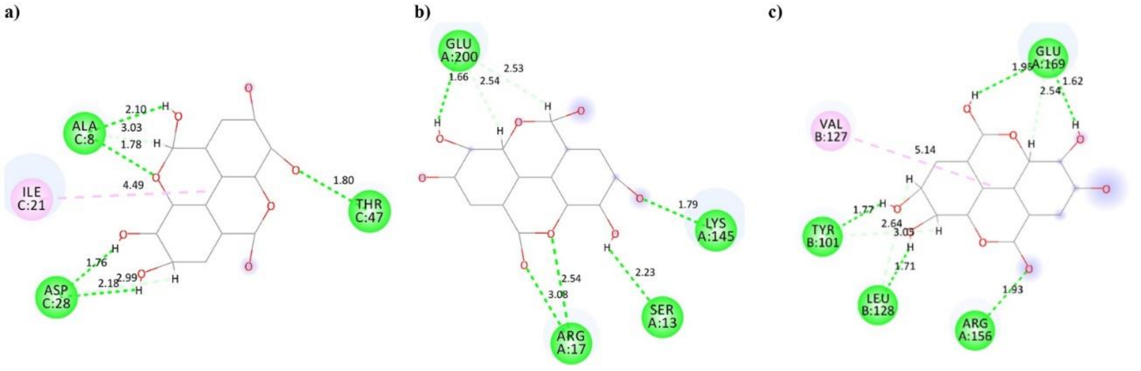

Figure S4: Diagram 2D of Interactions between ellagic acid and targets for antimicrobial activity: a) Dihydrofolate reductase (PDB ID 7K6C); b) Phosphoribosylaminoimidazole-succinocarboxamide synthase (PDB ID 6YYB), and c) tRNA (guanine-N(1)-)-methyltransferase (PDB ID 6QR4). Interactions: ■ - Conventional Hydrogen Bond, ■ – Alkyl, ■ - Carbon Hydrogen Bond.

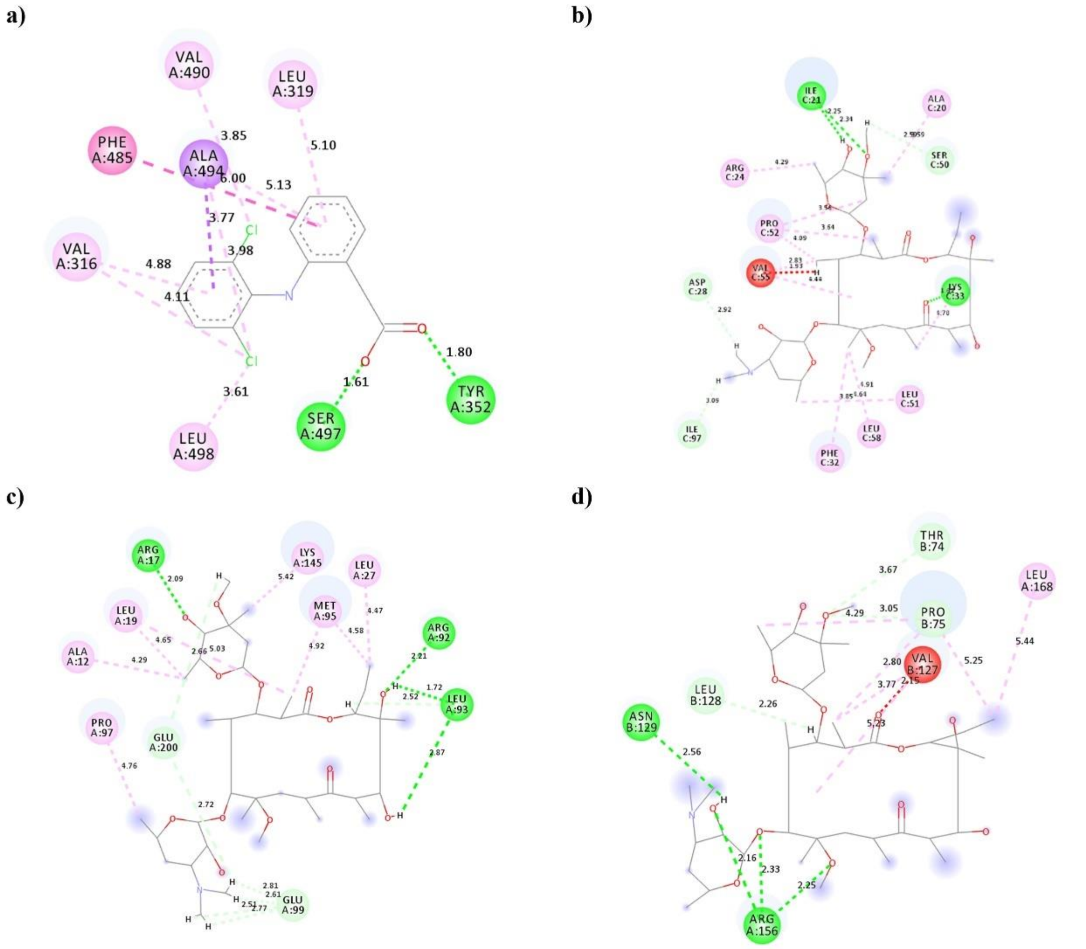

Figure S5: Diagram 2D of Interactions between: a) diclofenac and COX2 (PDB ID: 5IKQ); b) clarithromycin and Dihydrofolate reductase (PDB ID 7K6C); c) clarithromycin and Phosphoribosylaminoimidazole-succinocarboxamide synthase (PDB ID 6YYB), and d) clarithromycin and tRNA (guanine-N(1)-)-methyltransferase (PDB ID 6QR4).

Table S1: Predicting molecular properties related to Lipinski's rule

Compound	Oral Bioavailability (< 140 Å²)	MW (<500)	ALogP (≤5)	HBD (≤5)	HBA (≤10)
Clarithromycin	183.726	747.477	2.200	4	14
Diclofenac	50.926	269.149	4.373	2	3
Ellagic Acid	135.723	302.193	1.584	4	8

AlogP = the logarithm of the partition coefficient between n-octanol and water; MW: Molecular Weight; HBD: Hydrogen Bond Donnor; HBA: Hydrogen Bond Acceptor.
